# Microglial Phagocytosis of Neurons: Diminishing Neuronal Loss in Traumatic, Infectious, Inflammatory, and Autoimmune CNS Disorders

**DOI:** 10.3389/fpsyt.2019.00712

**Published:** 2019-10-03

**Authors:** Samuel F. Yanuck

**Affiliations:** Program on Integrative Medicine, Department of Physical Medicine and Rehabilitation, University of North Carolina School of Medicine, Chapel Hill, NC, United States

**Keywords:** depression, microglia, neuron, autoimmunity, inflammation, traumatic brain injury, cytokine, excitotoxicity

## Abstract

Errors in neuron-microglial interaction are known to lead to microglial phagocytosis of live neurons and excessive neuronal loss, potentially yielding poorer clinical outcomes. Factors that affect neuron-microglial interaction have the potential to influence the error rate. Clinical comorbidities that unfavorably impact neuron-microglial interaction may promote a higher rate of neuronal loss, to the detriment of patient outcome. This paper proposes that many common, clinically modifiable comorbidities have a common thread, in that they all influence neuron-microglial interactions. Comorbidities like traumatic brain injury, infection, stress, neuroinflammation, loss of neuronal metabolic integrity, poor growth factor status, and other factors, all have the potential to alter communication between neurons and microglia. When this occurs, microglial phagocytosis of live neurons can increase. In addition, microglia can shift into a morphological form in which they express major histocompatibility complex II (MHC-II), allowing them to function as antigen presenting cells that present neuronal debris as antigen to invading T cells. This can increase risk for the development of CNS autoimmunity, or can exacerbate existing CNS autoimmunity. The detrimental influence of these comorbidities has the potential to contribute to the mosaic of factors that determine patient outcome in some CNS pathologies that have neuropsychiatric involvement, including TBI and CNS disorders with autoimmune components, where excessive neuronal loss can yield poorer clinical outcomes. Recognition of the impact of these comorbidities may contribute to an understanding of the common clinical observation that many seemingly disparate factors contribute to the overall picture of case management and clinical outcome in these complex disorders. In a clinical setting, knowing how these comorbidities can influence neuron-microglial interaction can help focus surveillance and care on a broader group of potential therapeutic targets. Accordingly, an interest in the mechanisms underlying the influence of these factors on neuron-microglial interactions is appropriate. Neuron-microglial interaction is reviewed, and the various mechanisms by which these potential comorbidities influence neuro-microglial interaction are described.

## Introduction

Impaired neuronal function (1–4), from neuroinflammation or other causes, is a known driver of depression and related neuropsychiatric problems (5–11) that often accompany complex CNS disorders, including TBI (12, 13) and CNS disorders with autoimmune components (14), such as multiple sclerosis (MS) (15, 16), Parkinson’s disease (PD) (17–19), and Pediatric Acute-onset Neuropsychiatric Syndrome (PANS)/Pediatric Autoimmune Neuropsychiatric Disorders Associated with Streptococcal Infections (PANDAS) (20). A recent twin study by Huang, et al, suggests that the causal connection between depression and neuroinflammation may be bidirectional. (21)

Neuronal impairment can occur with insults to brain function from trauma, as with TBI (reviewed in 22), or can be metabolic or immunological, as with neuroinflammation (5–11), loss of neuronal cellular metabolic integrity [reviewed in Ref. (23)], infection [reviewed in Ref. (24)], or autoimmune response to neuronal targets.

Neuronal impairment can also occur due to dysfunction of neuronal autophagy, the mechanism by which neurons repair themselves *via* autodigestive recycling of damaged organelles and other cellular constituents (25). The association between impaired neuronal autophagy and Alzheimer’s disease (AD) has been reviewed by Uddin, et al. (26). The association between impaired autophagy and PD has been reviewed by Sheehan and Yue (27), and with neurodegeneration by Menzies (28). Plaza-Zabala et al, have proposed that the dysregulation of autophagy in microglia affects their capacity for phagocytosis and affects whether they adopt an inflammatory morphology, with implications for aging and neurodegenerative diseases (29). Yoshii and Mizushima have reviewed the practical impediments to measuring autophagy in humans (30).

Both traumatic and non-traumatic insults to brain can trigger mechanisms of ongoing neuronal destruction that are inherently immunological, as microglial cells phagocytize live but marginally functioning neurons (31). Details of the signaling mechanisms that instigate this process have been described by Fricker et al. (32) and by Neher et al. (33), cited in Galloway et al. (34).

Neuron – microglial signaling can be influenced by neuroinflammation, metabolic impairment, and microglial activation. In cases involving autoimmune processes, the clinical outcome also depends on appropriate ongoing immune modulation. In cases involving an infectious etiology, maintenance of a non-infected status may be crucial to favorable patient outcome. These processes are themselves influenced by a group of known comorbidities, whose biological influences have been described.

It is this author’s contention that, in a clinical setting, knowing how comorbidities that affect neuron-microglial signaling can influence patient biology may create the potential for clinical advantage in patients with neuropsychiatric disorders, through clinical attention to seemingly disparate factors like exercise, reducing inflammation, neurotransmitter (NT) support, stress reduction, and inhibition of autoimmune attack of self-tissue. While these clinical interventions involve many complex factors, they share an influence on the fundamentally convergent core mechanisms governing neuron-microglial interaction, some by supporting intact neuronal metabolism in support of adequate neuronal firing rate (frequency of action potentials) and NT release (exercise, NT support, glycemic control, oxygenation, etc.), some by reducing neuroinflammation (stress reduction, elimination of infection, etc.), some by other mechanisms. Understanding the details of neuron-microglial interactions may be of use in identifying specific factors involved in a given patient’s case and matching clinical interventions to specific targets that appear to be involved in dysregulating neuron-microglial interaction. This may help sharpen efforts to help patients with difficult neuroimmunological disorders, for whom prognosis is often suboptimal.

## Neuron-Microglial Interactions in the Healthy Brain

Many studies acknowledge the role of neuroinflammation as a causal factor in the biology of depression (5–11). Neuroinflammation has profound effects on neuron - microglial interactions. To better understand the clinical implications of neuron - microglial interactions and their application in neuropsychiatric disorders, we discuss here the biology of neuron - microglial interactions and the factors affecting these interactions that can be influenced in the clinical setting.

The origin and development of microglia have been reviewed in detail by Ginhoux et al. (35). Microglial cells are specialized macrophages (36). They are the predominant immune cells in the healthy brain (37). They arise from CD45+ bone marrow precursors that colonize the fetal brain, where they migrate from the periphery during fetal development. Parenchymal microglia are uncommitted myeloid progenitors of immature dendritic cells and macrophages (38). Once established in the CNS parenchyma, microglia are sustained by proliferation of resident progenitors, independent of blood cells (39). It has been estimated that 13% of the CNS white matter consists of microglial cells (40). The functional plasticity of microglial cells that allows them to carry out a broad repertoire of activities has been reviewed by Gomez-Nicola and Perry (41). Microglia perform continuous surveillance on neurons (34, 35, 42, 43). The pruning of synapses by microglia is an essential part of normal brain development (44).

Normal pruning of synapses during brain development involves the elimination of excess or inappropriate neuronal connections, in support of a properly organized brain (44). In adulthood, synaptic pruning is part of the neural plasticity involved in learning (45). Galván describes the synaptic pruning that occurs in brain development and that of adults as occurring on a continuum (45). These are normal processes, in contrast with the excessive microglial phagocytosis of live neurons that is a primary effect of the altered neuron-microglial signaling with which this paper chiefly concerns itself (31).

Errors yielding excessive or deficient synaptic pruning during childhood brain development have been associated with neurodevelopmental disorders, including autism, epilepsy, and schizophrenia (46). Stress and its potential to induce neuroinflammation has been discussed by O’Connor, et al, as playing a potentially damaging role in brain development, increasing susceptibility to behavioral disorders in the pediatric population (47). Kariuki et al. have shown that infection early in life is associated with impairment of multiple aspects of childhood development (48). A meta-analysis by Khandaker et al. has shown an association between childhood CNS viral infection and later development of schizophrenia, which they describe as involving either direct pathogen influences or influences of the inflammatory process associated with the infection (49). Miller et al. have shown an association between dyslexia, a developmental disorder, and later development of some variants of progressive aphasia, a form of dementia (50). Neurons are also continuously lost in the healthy adult brain. Among several functions provided by microglial surveillance is the elimination by phagocytosis of the apoptotic bodies of these dead and dying neurons. Sowell et al. state that “A decline in gray matter volume is prominent between adulthood and old age, whereas white matter volume increases between 19 and 40 years, after which it steadily declines” [Ref. (51), citing Refs. (52–54)]. The normal process of neuronal loss thus presents a requirement for phagocytosis and clearance of apoptotic neurons by microglia, so that the neurons do not progress into secondary necrosis, a state that is highly inflammatory (55). Just as stone is removed during the creation of a sculpture, the daily loss of neurons during brain development is a normal function, leading to the emergence of the healthy adult brain. This pattern of neuronal loss continues in the adult. If the rate of neuronal loss is modest, the adult brain can maintain healthy function.

However, at any age, microglial morphology can change, disrupting the healthy homeostatic balance between neurons and microglia, and leading to excessive neuroinflammation, triggering or promoting the progression of neuronal loss and brain-based autoimmune processes such as MS, PD, dementia, PANS/PANDAS, and chronic traumatic encephalopathy (CTE) (56–60).

### The Error Rate

There are billions of neurons in the brain, and a comparable number of microglial cells (61, 62). Microglia perform continuous surveillance on neurons (34, 35, 42, 43). Using *in vivo* two-photon imaging of the neocortex, Nimmerjahn et al. (43), observed that “microglial cells are highly active in their presumed resting state, continually surveying their microenvironment with extremely motile processes and protrusions.” Kierdorf, describing both Nimmerjahn (43) and another two photon imaging study by Davalos et al. (63), states that, “two-photon microscopy imaging revealed a strict territorial organization of microglia *in vivo*… the processes of each microglial cell are highly dynamic, and retract and extend to sample the surrounding CNS. The sampling is highly random and has a high turnover rate, such that each microglia can sample the CNS parenchyma once in a few hours.”

The presence of billions of neurons, a comparable number of microglia, and the observation that microglia are “continually surveying” neurons, with microglia surveying the CNS parenchyma once in a few hours, suggests a number of surveillance events per day that is also in the billions.

In each of these surveillance events, a microglial cell senses a neuron *via* multiple signals. The microglial cell will either phagocytize or be inhibited from phagocytizing the neuron, based on the balance of these activating and inhibiting signals (32–34, 63–65). As Brown and Neher have described, microglial cells can incorrectly phagocytize “stressed but viable neurons,” an event they have termed “phagoptosis,” citing evidence that suggests phagoptosis can contribute to neuronal loss during brain development, inflammation, ischemia and neurodegeneration. The number of times that microglial cells commit these errors in phagocytic execution is a function of all of the inputs that determine the extent of phagoptosis. The higher the error rate, the more live neurons are inappropriately phagocytized, yielding a greater expression of dysfunction [reviewed by Brown and Neher (31)]. In some patients, this may be expressed as neuronal loss leading to dementia, CTE, or other inflammatory disorders. In other patients, the chief feature may be an expression of autoimmune process, discussed below. In either case, the expression of dysfunction centers on the change in neuron-microglial interaction, the change in microglial morphology, and the interactions of these two processes.

## Biology of Neuron-Microglial Interactions

Neuron-microglial interaction is bidirectional and involves two key mechanisms. The first is the set of factors driven by neuronal membrane bound factors like phosphatidyl-serine (PS) and substances released by neurons, like cytokines, neurotransmitters, and other immunological factors. These factors determine the extent of microglial phagocytosis of neurons and determine other features of microglial morphology.

The second key mechanism regulated *via* neuron-microglial interaction is the expression of major histocompatibility complex II (MHC-II) on the surface of microglia. This step enables microglia to function as antigen-presenting cells (APCs) and potentially activate T cell immunity. In the normal brain, microglia induce apoptosis (programmed cell death) in T cells that enter the brain, *via* ligation of CD95, aka FAS (FS-7-associated surface antigen) (66).

Macrophages are capable of substantial plasticity and can change their physiology in response to environmental cues, yielding populations of cells with distinct functions, which can promote or suppress inflammation (36). Of particular interest is the transformation of a microglial cell from a phagocytic macrophage morphology to an APC morphology. Antigen presentation capability in the brain is provided by microglial cells, not by the dendritic cells that serve this function in the periphery (40). This capability is necessary for proper immune surveillance against pathogenic organisms in the brain. However, if the microglial cell presents fragments of neuronal debris as antigen to T cells that have invaded the brain, autoimmune process in the brain can be initiated or promoted.

### Microglia in the Healthy Brain

In the healthy brain, microglial cells phagocytize apoptotic neurons and prune synapses of live neurons in a way that promotes neuronal function and brain health (31). As is the case with macrophage phagocytosis of apoptotic cells in the periphery, phagocytosis of apoptotic neurons by microglia yields production and secretion of anti-inflammatory cytokines that are released into the local brain parenchyma (66). During neuronal repair, phagocytic microglia clean up debris from injured neurites and secrete neurotrophic substances, such as brain derived neurotrophic factor (BDNF), to enhance repair (67). Ayata et al. have recently found evidence that the polycomb repressive complex 2 (PRC2) epigenetically restricts the expression of genes that support microglial clearance of neurons in the striatum and cortex (68). Brown and Neher point out that the healthy brain is relatively free of invading T cells, which are kept out of the brain by the intact blood brain barrier (BBB). Brain inflammation, which occurs behind the BBB, therefore differs from inflammation in the periphery by the relative absence of leukocytes (including neutrophils, monocytes, B cells, and T cells) and antibodies (37).

In addition to the maintenance of a healthy non-inflammatory brain parenchymal environment, glial cells, along with neurons, express cellular death signals including CD95Fas/CD95L (aka FasL), tumor necrosis factor-related apoptosis-inducing ligand (TRAIL) and TNF receptor (TNFR). These cellular death signals trigger apoptosis of T cells and other infiltrating cells (66). In the healthy brain, microglia and astrocytes also express FasL, which also induces apoptosis in T cells that migrate from the periphery into the brain (69). Induction of T cell apoptosis is an important housekeeping mechanism by which microglial cells eliminate invading T cells that could otherwise participate in the autoimmune activation processes.

### Microglia in the Dysfunctioning Brain

The first activity of microglia that adversely affects neurons occurs when microglia are acting as phagocytic cells, engaging in inappropriate phagocytosis of neurons. Brown and Neher have shown that microglia can execute neuronal death by phagocytizing stressed-but-viable neurons, which they have termed “phagoptosis” (31). It is noteworthy that mast cell-microglial interactions can play an important role in microglial activity. This has been reviewed by Skaper et al. (70).

The second activity of microglia that adversely affects neurons occurs when microglia act as APCs, presenting neuronal debris as antigen to invading T cells. In inflammatory and neurodegenerative diseases, including MS, infections, trauma, stroke, neoplasia, and Alzheimer’s disease, glial cells such as microglia gain antigen-presenting capacity through the expression of MHC molecules (71).

Experimental autoimmune encephalomyelitis (EAE) is the mouse model used to study MS. In an EAE model, Ponomarev et al. found that microglial activation occurred before the onset of disease symptoms and infiltration of peripheral myeloid cells into the CNS, suggesting that resident glial cells were involved in the initiation of disease. They found further that activated microglial cells underwent proliferation and up-regulated the expression of CD45, MHC-II, CD40, CD86, and the dendritic cell marker CD11c. At the peak of EAE disease, activated microglial cells comprised 37% of the total macrophage and dendritic cell populations and co-localized with infiltrating leukocytes in inflammatory lesions (72).

MHC-II, CD40, and CD86 are all required for antigen presentation and co-activation in the signaling between microglial cells and T cells. Expression of these markers and of the dendritic cell marker CD11c, clearly demonstrates the shift in microglial morphology to a dendritic-cell-like APC morphology, in which presentation of neuronal debris as antigen can trigger or promote brain-based autoimmunity. Co-localization of invading T cells with microglia is a hallmark of microglial APC behavior (40). An antigen presentation capacity is necessary to the clearance of pathogens from the brain, and experimental inhibition of these functions yields catastrophic effects in animal models (73). Nonetheless, imbalance in this mechanism can yield up-regulation of neuro-autoimmunity (see **Table 1**).

**Table 1:** Microglial functions in the inflamed and non-inflamed brain.

Normal non-inflamed brain	Inflamed brain
Housekeeping microglial functions	Potentially problematic microglial functions
• Repair damaged neurons • Phagocytize apoptotic neurons • Inhibit inflammation • Induce apoptosis in invading T cells	• Phagocytize live but impaired neurons • Express MHC II • Present fragments of neuronal debris to invading T cells • Promote autoimmune process in brain

### Mechanisms of Neuron-Microglial Regulation

The key feature of healthy neuron microglial homeostasis is that microglial cells correctly identify and phagocytize only the neurons going through normal, apoptotic cell death. Viable neurons are left alone or repaired if necessary. Apoptosis of dying neurons is non-inflammatory (74).

In the healthy brain, regulation of this process is accomplished by signals produced by neurons that affect microglial cell behavior. However, changes in either neurological or immunological factors can disrupt this homeostasis.

### Neural Self-Control

Neurons control the behavior of microglial cells through a repertoire of signals, some membrane bound and some released into the surrounding environment. At the center of this moving multi-variable equation is phosphatidylserine (PS). PS in the normal cell sits on the inner wall of the cell membrane. When the cell undergoes apoptosis, PS is flipped to the outer cell membrane, where it is visible to other cells (32–34, 63, 65). This change signals macrophages that the cell is undergoing apoptosis, and is a candidate for phagocytosis by the macrophage. Throughout the body, macrophages perform surveillance on cells in the surrounding environment by palpating them. This is true of microglial surveillance of neurons as well. When neurons flip PS to their outer membrane, where it is then encountered *via* microglial cell palpation, the microglial cell is triggered to phagocytize the neuron. PS is normally confined to the inner cell membrane in intact neurons because the neurons continuously move it from the outer to the inner membrane using a group of phosphatidylserine translocases, which have been identified as the type 4 P-type ATPases (31).

In addition to PS and factors related to its recognition, other signal molecules affect neuron-microglial interaction. Biber et al. describe neuronal “Off” signals that keep microglia in their resting state and reduce pro-inflammatory activity, and “On” signals that are inducible, including purines, chemokines, and glutamate. Thus, Biber et al. describe neurons as key immune modulators in the brain (64).

Microglial uptake of pathogen associated molecular patterns (PAMPs) from pathogens in the brain or damage associated molecular patterns (DAMPs) from neuronal debris contributes to NFκB activation. Simultaneous activation of phagocyte NADPH oxidase (PHOX) and inducible nitric oxide synthase (iNOS) in microglia resulted in the disappearance of nitric oxide (NO), appearance of peroxynitrite and apoptosis. However, the chronic state of activation may progress to “resolution phase” where microglia are amoeboid, highly phagocytic, and produce anti-inflammatory cytokines (including IL-10 and TGFβ) in order to resolve the inflammation and clear up the debris (36).

In a healthy brain, the interplay of neurons and microglia is balanced. Healthy, viable neurons produce adequate “OFF” signals to repel microglial phagocytic interest. These include TGFβ, which inhibits effector T cell activation, neurotransmitters, which constitute a signal that a neuron is active, BDNF and other trophic factors, as well as factors that inhibit microglial inflammation and neurotoxicity. Neurons undergoing apoptosis in the normal brain produce “ON” signals, attracting microglial cells and promoting phagocytosis of the apoptotic neurons. These include glutamate that drives tumor necrosis factor alpha (TNFα) release and neuroexcitotoxicity, microglial chemoattractants like CCL21 and CXCL10, ATP that promotes chemoattraction and interleukin-1β (IL-1β) release, and others. When microglia phagocytize apoptotic neurons, they secrete TGFβ, which promotes a tolerogenic, anti-inflammatory tissue environment (74).

Normally, the non-phlogistic (non-inflammatory) microglial phagocytosis of apoptotic neurons involves engulfment and digestion of the neuron without release of additional neuronal debris into the tissue environment. The microglial cell produces anti-inflammatory cytokines that shift the surrounding tissue toward resolution of inflammation. If instead the neuron undergoes primary necrosis through direct or indirect trauma, or exposure to a pathogen or toxin, its death releases cell fragments and cytosolic contents into the tissue environment. The extracellular appearance of ATP and other intracellular contents triggers a pro-inflammatory response in surrounding microglia. In addition, incompletely digested/disassembled neuronal debris can be taken up by microglia and presented as antigen to invading T cells, promoting brain-based autoimmunity.

## Factors Affecting the Biochemistry of “On” and “Off” Signaling in Neurons and Microglia

Each of the factors described in this section affect the biochemistry of “On” and “Off” signaling through impacts on the underlying biology of neurons and microglial cells, shifting the function of neurons or microglia in a way that alters the overall sum of influences.

### Changes Affecting Outer Membrane PS Expression

Brown and Neher (31) describe several factors that can drive PS exposure, promoting apoptosis of neurons by microglia. These include ATP depletion, inhibition of phosphatidylserine translocases by oxidative stress, and increased calcium levels. All three of these conditions are commonly found in the clinical setting, including in patients with neuropsychiatric issues arising from head trauma, chronic neuroinflammation, or autoimmune disorders affecting brain. These biological processes represent potentially modifiable treatment targets.

Brown and Neher go on to assert that reversible phosphatidylserine exposure can occur in stressed-but-viable neurons as a result of non-toxic levels of glutamate, oxidative stress, or growth-factor withdrawal (31).

Brown and Neher state that phagocytosis of neurons with exposed phosphatidylserine can be mediated *via* several microglial receptors and opsonins, some of which are strongly upregulated by inflammation (31). This suggests again that modulation of brain inflammation represents a potentially modifiable treatment target. Brown and Neher state that, “…during inflammation, microglia and astrocytes release increased amounts of milk fat globule EGF factor 8 (MFG-E8; also known as lactadherin or SED1), which tightly binds to exposed phosphatidylserine through its C1 and C2 domains and to microglial vitronectin receptors (VNRs)—αvβ3 or αvβ5 integrins—through an RGD motif. The resulting activation of VNRs induces phagocytosis by activating a CRKII– DOCK180–RAC1 signaling pathway that causes remodeling of the microglial actin cytoskeleton.” They further state that, “On neurons, calreticulin exposure promotes their phagocytosis by binding to microglial low-density lipoprotein receptor-related protein (LRP), probably in association with other signals. In addition, the complement components C1q and C3, which are produced by microglia and astrocytes, may induce phagocytosis by binding to altered neuronal surfaces” (31).

Lunnon et al. have shown that systemic inflammation upregulates Fc receptors, including activating FcγRIII and FcγRIV, but not the inhibitory FcγRII. They conclude that, “systemic inflammation during chronic neurodegeneration increases the expression levels of activating FcγR on microglia and thereby lowers the signaling threshold for Ab-mediated cell activation” (75).

Huizinga et al. have shown that, in MS, fragments of axonal debris are taken up by microglial cells and degraded, a process that appears to support neuronal debris clearance, but that the authors describe as potentially playing a role in augmenting autoimmunity to neuronal antigens (76).

### Changes in Neuronal Metabolic Integrity Affecting Multiple Factors

Mitochondrial ATP production stands at the center of cellular function in all cells, including neurons. The success of the neuron in carrying out ATP production determines the success of all other cellular functions. If the neuron cannot produce ATP, it cannot continue to pump PS from its outer membrane to its inner membrane; nor can it produce adequate neurotransmitters, growth factors, or other “OFF” signals. If this occurs, the likelihood of apoptosis of live but metabolically compromised neurons is increased. This may increase the rate of phagocytosis errors, favoring more frequent microglial phagocytosis of live neurons whose ATP production is compromised (31).

Inflammation, oxidative stress, and persistently excessive production of epinephrine associated with stress have all been shown to be associated with mitochondrial dysfunction (77–79).

At the simplest level, neurons require stimulation, oxygen, and glucose. Impairment of any of these functions will diminish metabolic integrity.

Adequate glucose is required for the normal function of all cells, including neurons. Glycemic dysregulation in diabetes, hypoglycemia, insulin resistance and other such conditions can impair systemic and consequently CNS glucose levels. Many diabetic patients also have microcirculatory problems, compounding blood glucose issues with poor circulatory delivery of oxygen and glucose.

Likewise, CNS oxygen levels can be impaired by respiratory disorders like asthma and COPD, by cerebrovascular disorders, or by systemic microcirculatory disorders that also affect brain.

Fumagalli et al. reviewed changes that occur with hypoperfusion in the brain, such as can occur with stroke or TIA, leading to hypoxia, which causes excitotoxicity, oxidative stress, BBB dysfunction, microvascular injury, hemostatic activation, post-ischemic inflammation and neuronal death. Fumagalli et al. go on to say that these events contribute to changing the ischemic environment over time and, consequently, the behavior of microglia and macrophages (80).

### Changes Driving Brain Inflammation

Brain inflammation differs from inflammation in the periphery because of the relative absence of leukocytes and antibodies. There is a limited traffic across the BBB and this traffic can be increased by inflammation, which can recruit leukocytes into the brain (36).

**Figure 1:**
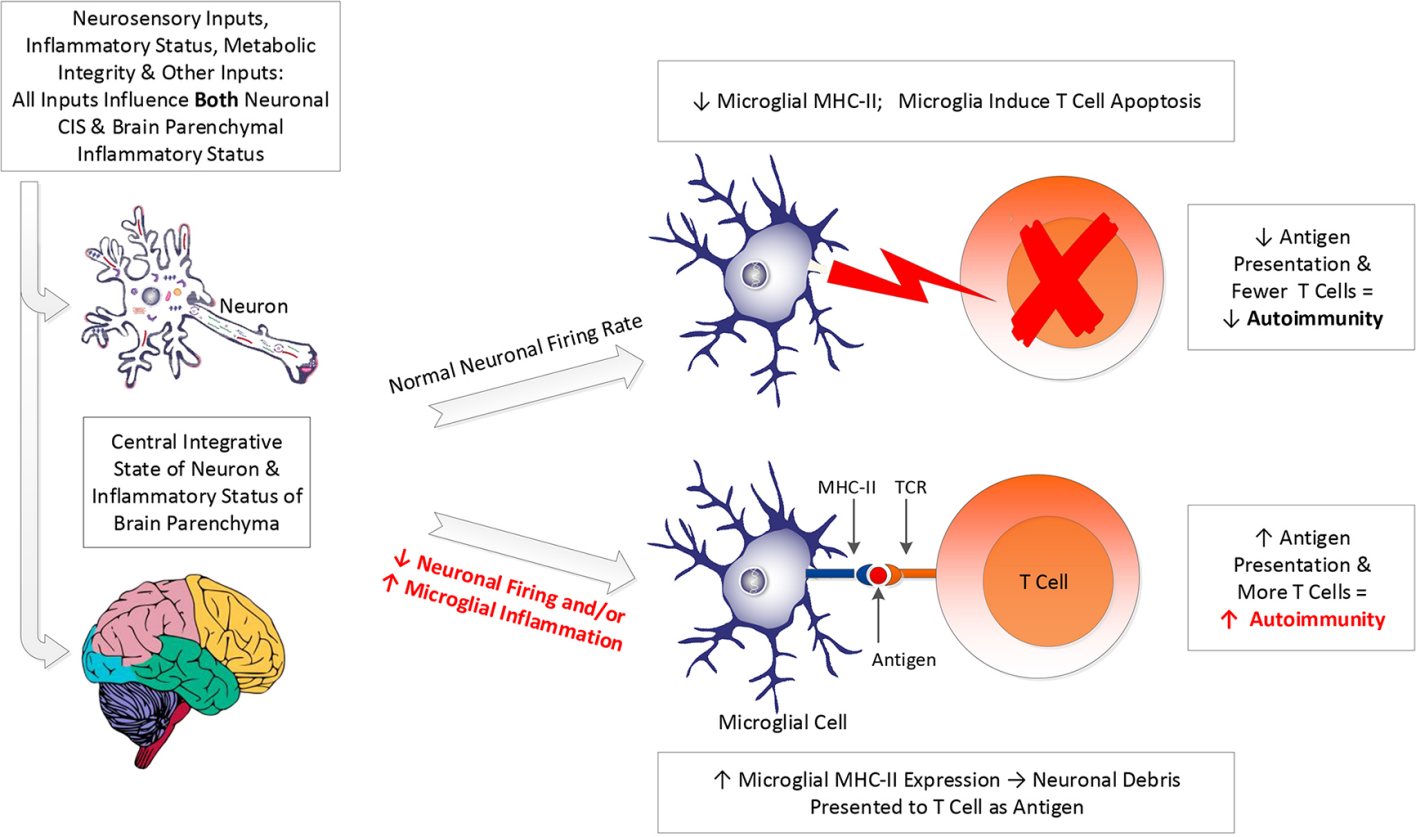
Impact of neuronal CIS and inflammation on microglial function. A host of inputs determine both the central integrative state (CIS) of neurons and the extent of neuroinflammation (see text). These two factors determine whether the neuronal firing rate (frequency of action potentials) will be rapid enough to generate the electrical activity necessary to inhibit microglial expression of major histocompatibility complex II (MHC-II) on their cell membranes. If neuronal electrical activity is adequate, microglial expression of MHC-II will be inhibited, and microglia will be prevented from presenting antigen to T cells. Instead, microglia will be kept in their resting/ramified state, in which they induce apoptosis in T cells that enter the brain, *via* FAS/FASL interactions. This diminishes the risk of expression of autoimmune process in the brain. If neuronal signaling is inadequate, or if neuroinflammation is increased, it becomes more likely that microglia will express MHC-II and will perform antigen presentation of neuronal debris to the T cell receptors (TCR) of invading T cells. This increases the risk of developing autoimmune process with neuronal tissue as the target of the autoimmune process.

As discussed, brain inflammation induces unfavorable changes to microglial morphology, yielding a state in which microglia phagocytize viable neurons, express MHC-II, and present fragments of neuronal debris to T cells instead of inducing the T cells to undergo apoptosis. This is in contrast to the normal brain, in which microglia induce apoptosis of invading T cells *via* FAS/FASL interactions (66). The result of the inflammation-induced shift in microglial morphology and behavior can be excessive neuronal loss and upregulation of brain autoimmunity (66) (see **Figure 1**).

Inflammation also causes impaired neuronal metabolic integrity. It is noteworthy that TNFα and IL-1β, two important “ON” signals, are both primary pro-inflammatory cytokines that are upregulated in the context of brain inflammation. The theme of neuroinflammation driving deleterious effects in brain suggests the importance of identifying and addressing causes of brain inflammation in the clinical setting.

One of the primary drivers of brain inflammation is body inflammation (81). Increases in pro-inflammatory cytokines in the periphery yield upregulation of brain inflammation and potentiate neuronal death (71, 82–84). Systemic IL-1β can cause CNS inflammation once it enters the brain, thus linking systemic inflammation and immune activation with brain inflammation (56).

Perry et al. state that microglial phenotype is modified by systemic infection or inflammation. They go on to say that the fact that diseases with a chronic systemic inflammatory component are risk factors for AD implies that crosstalk occurs between systemic inflammation and microglia in the CNS (85). AD risk has been shown to be increased in patients with metabolic syndrome and adiposity (86), decreased diversity of the intestinal microbiome (87), periodontal disease (88), infection (89), and vascular risk factors like type 2 diabetes, smoking, hypertension and heart disease (90), and stroke (91).

With regard to arthritides, Fusco et al. have reviewed contributions to neuroinflammation from osteoarthritis and rheumatoid arthritis (RA), suggesting contributions from both *via* upregulation of IL-1β and mast cell mediated microglial activation (92). In patients with RA, neuroinflammation, and AD, Schrepf et al. using multimodal MRI, have demonstrated patterns of CNS connectivity that predicted fatigue, pain and cognitive dysfunction during phases of RA flare (93). Chou et al. in a case control trial of 8.5 million patient records from the US, Puerto Rico, and the USVI, found that AD was more prevalent among RA patients (0.79%) than among those without RA (0.11%), that relative AD risk was increased by the presence of comorbidities like diabetes and peripheral vascular disease, and that relative AD risk was lowered by etanercept, but not by the other anti-TNF agents studied or other immunosuppressive medications (94). Cao et al. analyzing a Taiwanese patient database, found an inverse association between prior RA and AD (95). Contrastingly, Policicchio et al. in a meta-analysis and Mendelian randomization study, found that an apparent inverse correlation between RA and AD identified in previous studies is not causal (96). Cai et al. analyzing a large GWAS dataset of patients of European descent, also found no association between RA and AD (97).

van Langenberg et al. in a human trial, found that patients with Crohn’s disease had cognitive impairment attributable to systemic inflammation (98). McCaulley and Grush have reviewed evidence for the clinical utility of TNFα inhibitors in AD, noting that etanercept has shown a clinical benefit. They point out that etanercept has not shown benefit in inflammatory bowel disease (99).

Ajami et al. describe two distinct pools of monocytic cells in MS and EAE: infiltrating monocytes and resident microglial cells. They observed a strong correlation between monocyte infiltration from the periphery into the brain and progression to the paralytic stage of EAE. They go on to state that increases in circulating inflammatory monocytes have been shown to correlate with relapses in EAE mice (100).

Lipopolysaccharide (LPS), aka endotoxin, is a PAMP found in the outer membrane of gram-negative bacteria that is a known B cell mitogen. Increasing the peripheral LPS level can induce the activation of central pro-inflammatory mechanisms, even when the amount of LPS used for stimulation is minimal or when the peripheral inflammatory cytokine levels are suppressed artificially. Both central and peripheral inflammation can exacerbate local brain inflammation and neuronal death (101).

Rivest (73) citing others (102–105) observed “a single systemic injection of LPS (1 mg/kg intraperitoneally) results in the robust induction of expression in microglial cells of genes that encode pro-inflammatory cytokines and chemokines, as well as proteins of the complement system” (73). This suggests that a bodily infection with gram negative bacteria can drive brain inflammation (73).

Other authors have also discussed the impact of factors involved in systemic inflammation on CNS inflammation. LaFlamme et al. (106) showed that, in a mouse model of sterile inflammation but not in endotoxemia, IL-1β is necessary for the activation of NF-kappaB and prostaglandins in the endothelial cells of the BBB.

Varatharaj and Galea (107) have reviewed mechanisms by which both sterile and infectious systemic inflammation can affect the BBB, with inflammatory consequences in the CNS, reflected in sickness behavior and other factors. They describe a heightened sensitivity of the diseased BBB to systemic inflammation, and a greater progression of AD and MS in patients with systemic inflammation. Kuhlmann et al. have described mechanisms by which c-reactive protein (CRP) promotes degradation of BBB integrity (108), though mention of CRP is notably absent in Varatharaj and Galea (107). Sproston and Ashworth, describe CRP as associated with both inflammation and infection (109).

Banks et al. have discussed the transport of IL-1β, a microglial “ON” signaling molecule, across the BBB in sickness behavior (110). Lin et al. (111) showed in a human trial that increased serum oxidative stress was associated with declined perceptual functioning in patients with PD. A meta-analysis by Peng et al. (112) found that post-operative cognitive dysfunction was correlated with the concentrations of peripheral inflammatory markers, particularly interleukin-6 and S-100β.

Denes et al. (113) in a mouse middle cerebral artery occlusion (MCAo) model of stroke, found that both LPS and anaphylaxis (induced by ovalbumin exposure in sensitized mice) induced inflammatory changes in the blood and in the brain prior to induction of stroke. After MCAo, both LPS and anaphylaxis increased microglial interleukin-1a (IL-1a) expression and blood-brain barrier breakdown, with profound impairment of survival in the first 24 h after stroke in the presence of systemic inflammatory challenges. They concluded that these finding “suggest that systemic inflammatory conditions induce cerebrovascular inflammation,” and that “increased brain inflammation, BBB injury and brain edema formation can be major contributors to impaired outcome in mice after experimental stroke with systemic inflammatory stimuli, independently of infarct size.”

Taken together, the work of these authors suggests a connection between factors contributing to systemic inflammation and the promotion of neuroinflammation. In the clinical setting, targeting biological factors outside the CNS that have been shown to upregulate neuroinflammation may present an opportunity to influence outcomes in cases involving neuroinflammation. Particularly in cases where other approaches have not yielded improvement, further study of a clinical inventory of factors that have the potential to impact systemic inflammation may be warranted.

Infection (73, 114, 115), stress and depression (6, 116–118), altered microbiota (120) and other forms of digestive dysregulation (119–122), dysglycemia, insulin resistance, and obesity (123–126), tissue hypoxia [80, 127(review), 128, 129], poor diet (130–134), lack of exercise (135–139), environmental or other toxic exposure (140–144), and autoimmune processes (145, 146) are all potential drivers of body inflammation. As such, these factors may be worthy of attention in patients with neuropsychiatric disorders associated with neuroinflammation, patients with head trauma, neuroinflammation, or any condition known to be worsened by neuroinflammation and changes in microglial activation, including PD, AD or other dementias, ALS, MS, and PANS/PANDAS.

Bredesen et al. report significant reversal of cognitive decline, including some patients who returned to work, in a series of patients diagnosed with AD, assessed *via* quantitative MRI and neuropsychological testing. The approach, described as personalized rather than monotherapeutic, focused on identifying and modifying factors like neuroinflammation, infection, oxidative stress, dysglycemia, hypoxia and poor diet (147). The effect of the MS medication glatiramer acetate is partly attributed to its ability to modify microglial morphology and the resulting impacts on neuron-microglial interactions (148). Feinstein et al. have reviewed treatment approaches focused on restoration of noradrenaline to influence both neurons and microglia (149). Other human studies (150–153) and reviews (154–156) have described improvements in neurological function using clinical approaches that target oxidative stress, BDNF, and neuroinflammation. Regarding biomarkers, Hirad et al. have demonstrated that reduced midbrain white matter integrity, demonstrated on MRI, correlates with post-concussion deficit severity and with serum tau levels, a marker of blood-brain barrier disruption (157). Strawbridge, et al, have reviewed biomarkers of depression (158). Though some biomarkers related to neuron-microglial signaling, such as serum TGF beta, IL-6 or TNFα levels, or the extent of midbrain white matter loss observed on MRI, are obtainable in a clinical setting, most of the markers involved in direct measurement of the mechanisms described in research on neuron-microglial signaling interactions are not available to the clinician.

It is noteworthy that there are also mechanisms by which disorders whose primary locus of dysfunction is in the CNS can impact systemic inflammation, such that the arrow of causation does not always point from the periphery to the CNS. For example, efferent neuronal signaling from the dorsal motor nucleus (DMN) of the vagus nerve is known to inhibit sympathetic outflow (159) and to inhibit the production of inflammatory cytokines in the spleen (TNFα), liver (IL-6), and small intestine (TNFα) (160–162). Support for vagus nerve motor activity has been shown to inhibit kidney damage in an ischemia-reperfusion model (163). Any CNS disorder that involves diminished DMN signaling thus has the potential to influence the patient toward increased systemic inflammation.

## Microglial Priming

The pattern of inflammatory activation of microglia has been referred to as microglial priming. The hallmark of primed microglia is a shift from the resting or ramified state to one in which they swell and fill with pro-inflammatory cytokines (55, 67, 164, 165). Priming can be induced by aging, trauma, infection, or other stimuli. Microglia can remain in the primed state for long periods of time, without returning to the ramified state, but without releasing their bolus of cytokines. In a clinical setting, the concern is that any new trauma or insult introduced into a brain containing primed microglia will result in a flooding release of pro-inflammatory cytokines that can be damaging to the brain (55, 67, 164, 165). The first injury shifts microglia into the primed state. When the second TBI or non-traumatic brain insult occurs, the microglia release a flood of pro-inflammatory cytokines, yielding more deleterious effects. This is the basis for the common observation that a second or subsequent traumatic brain injury often yields more serious consequences and carries the potential for a poorer prognosis.

Since systemic inflammation can drive brain inflammation, factors capable of driving body inflammation predispose the microglia to a shift into the primed state. If body inflammation and thus brain inflammation is already present at the time of a new brain insult, whether that new insult is a TBI, or a biochemical insult associated with autoimmune process, metabolic insult, infection, or other factors, the prognosis is likely to be worse.

This suggests that, for individuals already affected by neuropsychiatric disorders, neuro-autoimmunity, or an existing head trauma, there may be utility in taking steps to prevent systemic and neuroinflammation, oxidative stress, loss of metabolic integrity, and related factors, with the twin goals of diminishing existing neuronal impairment and protecting against the eventuality of additional insults.

It is especially noteworthy that a new brain insult can result from causes other than trauma. As referenced above, upregulation of inflammation can occur from a new infection in the body, an upsurge of stress due to a change in life circumstance, overtraining syndrome, glycemic dysregulation, immunological reactions to environmental factors like chemicals, pollens, pollutants or other antigenic burdens, or a host of other instigating factors. Any of these factors, if they induce neuroinflammation and affect microglial function sufficiently, can instigate a flooding release of inflammatory mediators into the brain parenchyma, resulting in a worsening of symptoms. Introduction of any of these comorbidities can change the ongoing equation in the brain in a way that can tip the balance of neuron-microglial interaction toward a higher error rate or, in more severe cases, constitute a biological second hit. This “two hit” or “multiple hit” theory has been described in TBI (67, 166, 167) and in non-traumatic forms of brain insult, including aging (164, 167), neurodegenerative diseases (167–170), and perinatal white matter injury (171).

Thus, clinical measures taken to reduce systemic inflammation accomplish the triple agenda of reducing current impairment, reducing the potential effect of a second hit from actual trauma (like head banging in autism or sport-related impact), and reducing the risk of a second hit effect occurring from a non-traumatic pro-inflammatory insult.

## Electrical Cell-Signaling Effects

### The Role of Neuronal Electrical Activity in Maintenance of Neuron-Microglial Homeostasis

Microglial antigen presentation capability depends on microglial MHC-II expression. As Neumann points out; the electrical activity of healthy neurons suppresses MHC-II expression in surrounding glial cells. Hence, the antigen presentation process that leads to autoimmunity is counter-regulated in intact CNS areas by the electrical activity of neurons (101). Similarly, Grieb et al. observed that when high frequency electrical stimulation was applied to the subthalamic nucleus of freely moving animals, gene enrichment analysis showed the strongest regulation occurred in MHC-encoding genes (172).

The repertoire of neuronal control mechanisms regulating microglial function thus includes both biochemical factors (neurotransmitters and other “Off” signals produced as a consequence of normal neurotransmission) and neuronal-firing-rate-dependent electrical inhibition of microglial MHC-II expression.

This implies that there are two distinct immune consequences to reductions in neuronal firing rate. These are: diminished production of NT’s and other “OFF” signals, and a reduction in electrical activity that allows greater microglial MHC-II expression. The microglia in this situation are not adequately inhibited from phagocytizing live neurons (31, 64), nor are they adequately inhibited from functioning as APCs (101, 172).

The capacity of neurons to maintain robust firing rate depends in large part on two factors. The first, metabolic integrity of the neuron (the neuron as a cell) is discussed above. The second is the central integrative state (CIS) of the neuron, which depends on the sum of its excitatory and inhibitory presynaptic stimuli. CIS is the sum of all activity within the neuron, partly resulting from external forces and synaptic signaling. CIS can be summarized as the combination of two factors: how close the neuron is to the firing threshold and how much metabolic integrity the neuron has, with which to maintain firing before fatigue.

Loss of neuronal firing rate will at some point yield a loss of the neuron’s ability to generate enough electrical activity to adequately inhibit microglial MHC-II expression in microglial cells affected by that neuron’s contribution to the electrical environment within the local brain parenchyma. This can occur as a consequence of impaired neuronal metabolic integrity, such that the neuron cannot generate enough ATP to sustain its firing rate. Or this can occur through direct neuronal injury, as with a TBI, or with diminished presynaptic stimulation from whatever area of the brain normally fires into the neuronal pool in question.

Afferent stimulation from the body to the brain plays a significant role in the maintenance of the CIS of CNS neurons. Thus, factors like lack of exercise, destruction of joint mechanoreceptors in arthritic conditions, poor muscular tone, diminished rib movement with respiratory disorders and other such changes can alter the neurosensory environment, altering the CIS of neurons. This process of reduced CIS in initially uninjured brain areas due to processes conceptually similar to dysafferentation or deafferentation coming from truly damaged areas has historically been called focal or non-focal diaschisis based on distance and connectivity (173, 174). Carrera and Tononi define diaschisis as a change in structural and functional connectivity between brain areas distant to a lesion (173).

Discrete areas of the brain more affected by these or other factors will have relatively greater reductions in the firing rates of their neurons, yielding a reduction in electrically mediated inhibition of microglial MHC-II expression on the surface of microglia doing surveillance on those neurons. Accordingly, areas of the brain more affected by factors that lower neuronal firing rates will be more vulnerable to the expression of autoimmune processes. As Neumann states, “in a variety of inflammatory and neurodegenerative diseases, including MS, infections, trauma, stroke, neoplasia, and AD, glial cells such as microglia gain antigen-presenting capacity through the expression of MHC molecules. Further, proinflammatory cytokines, such as TNF, IL-1β, and IFNγ, as well as chemokines, are synthesized by resident brain cells and T lymphocytes invading the affected brain tissue… The pro-inflammatory cytokines stimulate microglial MHC expression in the lesioned CNS areas only” (71).

## More Inflammation Yields Further Consequences

The damage to neurons that results from autoimmune attack yields the release of pro-inflammatory DAMPS. This yields two effects that can be considered as occurring in a second wave of effects:

First, the increased levels of neuroinflammation will include increased levels of TNFα and IL-1β. These cytokines are neuronal “ON” signals, influencing neuron-microglial signaling in favor of the likelihood of additional microglial phagocytosis of live neurons (64).

Second, increased inflammation further impairs neuronal metabolic integrity, further diminishing the capacity of neuronal mitochondria to make ATP, further diminishing the capacity of neurons to sustain their firing rates. Loss of neuronal firing rates allows further microglial MHC-II expression. While this may appear to simply be a restating of the mechanism by which new brain autoimmunity can develop (40, 56–60, 71, 72), the presence of additional neuronal tissue debris, which can be taken up by microglia and presented as antigen, risks the development of distinct antibody reactions to new neuronal tissue epitopes, an effect known as epitope spreading (175, 176). Munz et al. define epitope spreading as, “… a mechanism by which an immune response that is initiated by various stimuli, including microbial infection, trauma, transplanted tissue or autoimmunity, ‘spreads’ to include responses directed against a different portion of the same protein (intramolecular spreading) or a different protein (intermolecular spreading)” (177).

**Figure 2:**
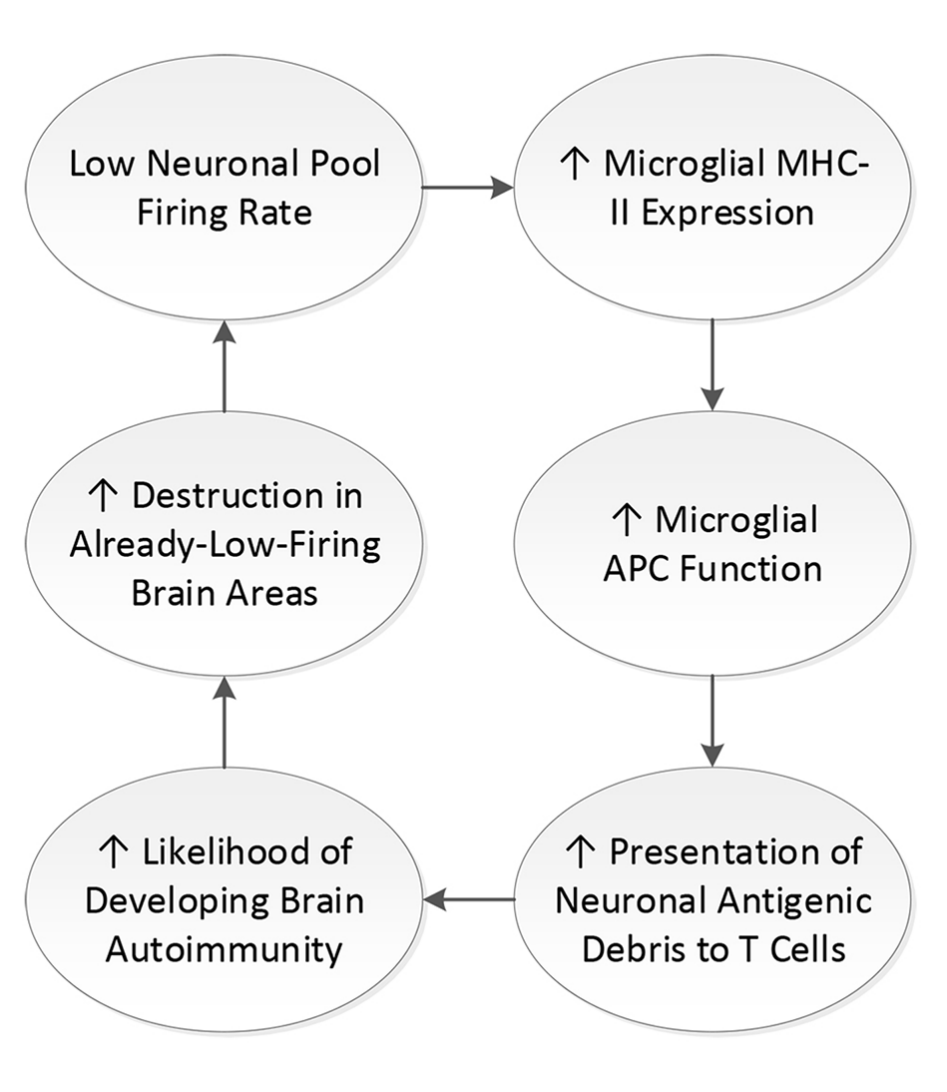
Feedback loop of low neuronal firing rate. When neuronal firing rate is diminished, microglial major histocompatibility complex II (MHC-II) expression is increased. MHC-II is the cell surface mechanism by which microglial cells present fragments of neuronal debris as antigen to T cells. Presentation of neuronal debris as antigen increases the likelihood of expression of brain autoimmunity, which creates destruction of neurons and increases neuroinflammation of those neurons that are not destroyed. Neuroinflammation diminishes neuronal firing rate, yielding a loop.

Thus, the loss of neuronal firing rates, itself a consequence of the inflammatory damage that occurs with brain insults, predisposes the affected brain areas to the development of new microglial antigen presentation events and opportunities for either new autoimmune process when the initial brain insult does not already involve autoimmunity (177), or epitope spreading and worsening of autoimmune-mediated damage in existing autoimmune brain disorders (177) (see **Figure 2**).

### Still More Consequences

When an autoimmune brain disorder, PD or MS, for example, is already present, there are two important clinical implications:

First, the introduction of a new event or process that increases neuroinflammation can trigger a worsening of the pre-existing autoimmune disorder, through both accelerated neuronal loss and epitope spreading. This suggests that, in cases involving brain based autoimmune diseases, it’s useful for clinicians to be particularly persistent in their attention to drivers of inflammation, including infection, stress, mood issues, digestive dysregulation, dysglycemia, hypoxia, dietary factors, immune responses to foods, haptens and environmental triggers, and other such factors. Clinical steps to mitigate these factors, and their pro-inflammatory effects, may be worthy of consideration.

**Figure 3:**
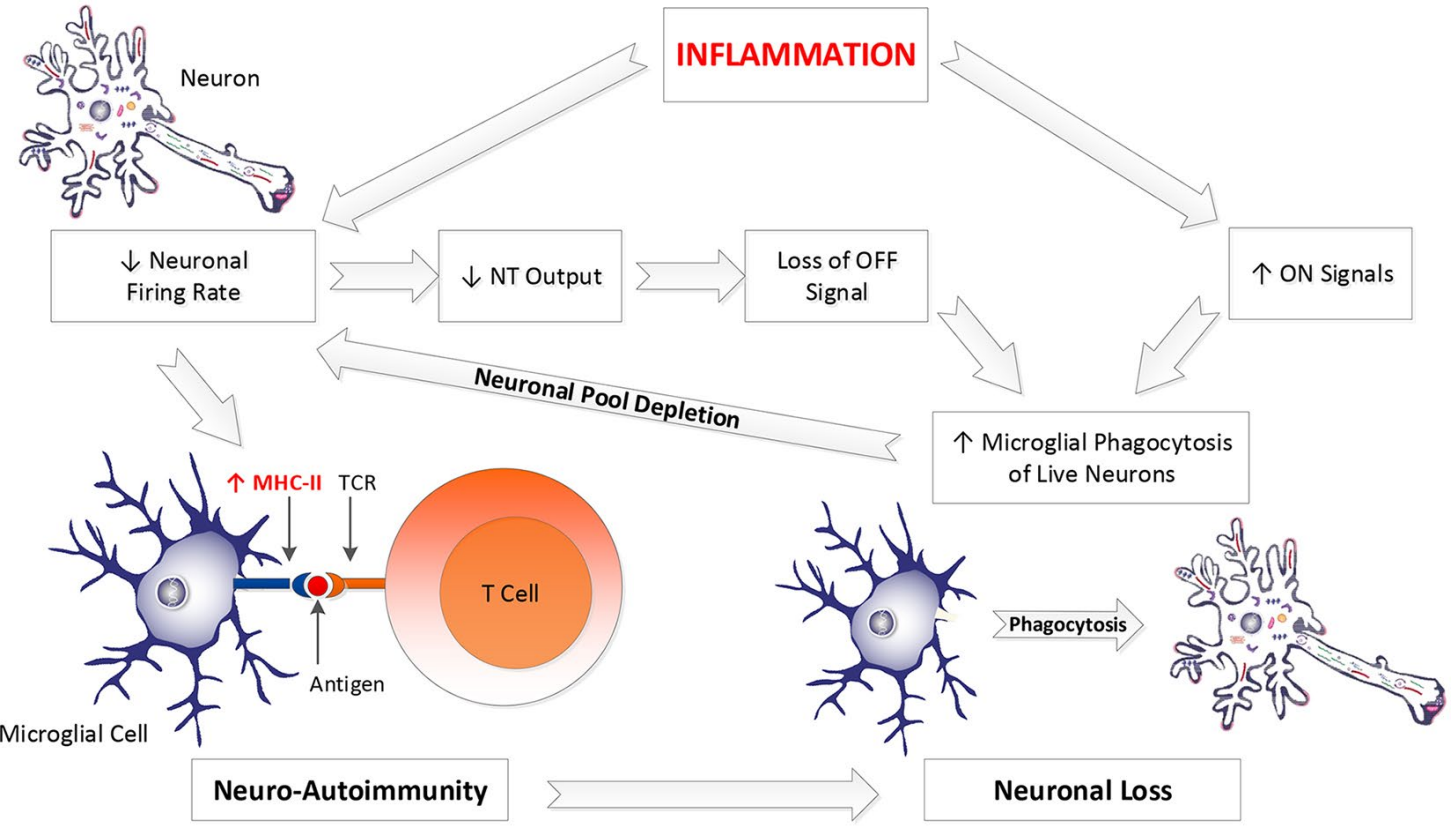
Inflammation drives both neuro-autoimmunity and excessive neuronal loss. Inflammation impairs neuronal firing rate, allowing microglial MHC-II expression, yielding presentation of neuronal antigenic debris to T cells, driving neuro-autoimmunity. Decreased neuronal firing rate also decreases neuronal NT output. Since NT’s are a significant OFF signal, diminished NT production constitutes the loss of an OFF signal. IL-1β and TNFα, two inflammatory cytokines, are part of the ON signal milieu. Loss of NT output and increased IL-1β and TNFα yield a tissue environment more favorable to microglial phagocytosis of live neurons. Depletion of neurons from a neuronal pool yields diminished electrical activity in that pool and greater vulnerability to MHC-II expression by microglial cells performing surveillance on those neurons. Hence, inflammation and subsequent loss of neurons increases the likelihood of developing subsequent neuro-autoimmunity. This promotes further neuronal loss. NT, neurotransmitter; MHC-II, major histocompatibility complex II; TCR, T cell receptor.

Second, the introduction of either abrupt or evolving diaschisis, resulting in loss of neuronal firing rates, might increase the risk of further microglial antigen presentation, development of epitope spreading, worsening of existing disease, and expansion of involvement in areas most affected by the diaschisis (see **Figure 3**).

Clinicians must be alert to changes that might trigger an evolving diaschisis. These might include changes in the patient’s capacity for voluntary movement, whether driven by pain, mechanical restriction, or changes in energy level; changes in respiratory patterns, or changes in the character of a tremor. Clinical steps to mitigate these factors, and to restore appropriate neuronal firing rates and CIS to affected central nervous system areas, are worthy of consideration. The proper clinical approach would depend upon the individual case. The aim in all cases would be to identify the change in afferent barrage to the area of diaschisis and to restore normal afferent stimulation to that area, with the goal of restoration of the normal central integrative state. Examples would include appropriate post-stroke rehabilitation, post-concussion rehabilitation, or restoration of afferent barrage of a CNS target from a hip, shoulder, or other sensory source after an injury or other dysfunctional change has taken place.

## Further Neuroimmune Triggers

### Pathogens or Damaged Neuronal Debris Evoke Excitotoxicity and Neuronal Cell Death

**Figure 4:**
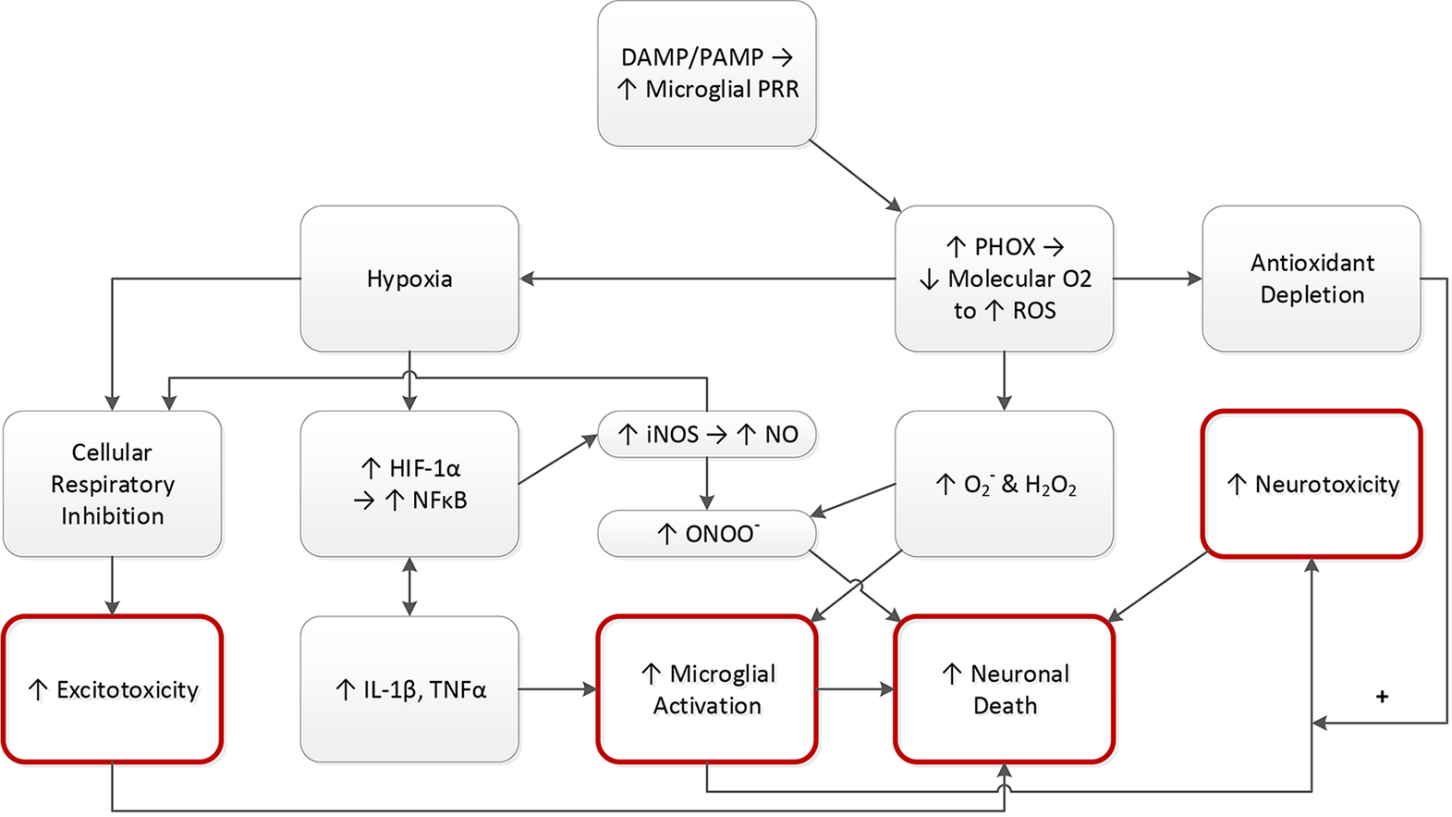
PAMPs and DAMPs drive neuronal destruction. DAMPs and/or PAMPs trigger PRR’s on microglia. Microglia generate PHOX, driving down O_2_ levels, as the O_2_ is consumed to make ROS. This yields three consequences: hypoxia, increased ROS (O_2_− and H_2_O_2_), and antioxidant depletion as a result of the increased ROS. Hypoxia raises the level of hypoxia inducible factor 1 alpha (HIF-1α), a substance that induces the formation of blood vessels in areas of inadequate oxygen. HIF-1α upregulates inflammatory NFκB, which induces gene expression of IL-1β and TNFα. Thus, hypoxia drives microglial activation, promoting neuronal death. NFκB upregulation also promotes activation of NO *via* iNOS induction. NO drives up ONOO−, which combines with the ROS to activate microglia, again promoting neuronal death. NO also combines with hypoxia to induce excitotoxicity, promoting neuronal death. Microglial activation also increases neurotoxicity, *via* production of glutamate and other substances, an effect that may be amplified by antioxidant depletion. Red circles connote key downstream effects. DAMP, damage associated molecular pattern; PAMP, pathogen associated molecular pattern; PRR, pattern recognition receptor; PHOX, phagocyte NADPH oxidase; O_2_, oxygen; ROS, reactive oxygen species; O_2_−, singlet oxygen; H_2_O_2_, hydrogen peroxide; HIF-1α, hypoxia inducible factor 1 alpha; iNOS, inducible nitric oxide synthase; NO, nitric oxide; ONOO−, peroxynitrite; NFκB, nuclear factor kappa B; IL-1β, interleukin 1 beta; TNFα, tumor necrosis factor alpha.

Pathogens in tissue create pathogen-associated molecular patterns (PAMPs) (178). Damaged tissue creates damage associated molecular patterns (DAMPs) (179). As specialized macrophages, microglial cell membranes contain pattern recognition receptors (PRRs) that sense PAMPS and DAMPS, triggering phagocyte NADPH oxidase (PHOX), which turns molecular oxygen into reactive oxygen species (ROS). This conversion depletes the tissue of molecular oxygen, yielding hypoxia, driving hypoxia inducible factor 1α (HIF-1α). Under normal circumstances, HIF-1α induction by hypoxia promotes useful effects like the growth of blood vessels in hypoxic tissue (180, 181), as well as the activation of neutrophils that have migrated into tissue (182). However, HIF-1α also up regulates NFκB (182, 183), causing gene expression of IL-1β and TNFα (184). Since they are neuronal “ON” signals, increased IL-1β and TNFα promotes the activation of microglial phagocytosis of neurons (64). IL-1β and TNFα activation also promotes further NFκB upregulation, with this loop activation originally described by Barnes and Karin as the “amplifying loop” of the inflammatory process (184). NFκB induces iNOS expression, yielding abundant nitric oxide (NO) production (43). Though NO can be cytoprotective (43), the combination of NO and hypoxia impair cellular respiration (43), yielding excitotoxicity and resulting neuronal death (185). The formation of ROS like singlet oxygen (O_2_^-^) and hydrogen peroxide (H_2_O_2_) drives microglial activation (43). O_2_^-^ and H_2_O_2_ in combination with NO yields peroxynitrite (ONOO^-^), driving neuronal apoptotic cell death (43). Activated microglia release neurotoxic factors like glutamate, TNFα, and FASL, increasing neurotoxicity and increasing the likelihood of neuronal cell death (43). Nocella et al. have suggested that, in athletes, antioxidant depletion from single nucleotide polymorphisms or other factors may have an impact on the long-term risk for developing neurodegenerative disease (186) (see **Figure 4**).

Block et al. state that microglial activation can occur through recognition of inflammatory signals such as LPS from gram-negative bacteria, causing the microglia to produce neurotoxic pro-inflammatory mediators. Microglia can also be activated by debris from neuronal damage (DAMPs), yielding reactive microgliosis, which is then toxic to neighboring neurons, which they describe as resulting in a perpetuating cycle of neuron death. Block et al. describe microgliosis as a “potential underlying mechanism of progressive neuron damage across numerous neurodegenerative diseases, regardless of the instigating stimuli” (56).

## Other Views and Questions Going Forward

The history of research on microglial cells has not been without controversy. Disagreement regarding the embryologic origin of microglial cells, now settled, has been reviewed by Ginhoux and Prinz (187). The evolving view of the role of microglia as villains or protectors, has been reviewed by Fernandez et al. (188). What has emerged is a view of microglial cells that is more complex, with the potential for both supportive and deleterious functions, depending upon a complex array of factors.

That neurons and microglial cells interact, that key components of these interactions are understood, and that these interactions are relevant biologically, appear to be supported by a substantial body of evidence. However, the clinical relevance of these neuroimmunological processes and the extent of their universality in patient populations are important questions that broaden our understanding and improve our perspectives for future study.

With regard to the effect size and clinical relevance of neuron-microglial interactions, the following papers discuss other relevant processes that precede neuron-microglial signaling and may play important roles: Culmsee et al. discuss the role of immunometabolic factors that influence mitochondria, with the potential to substantially alter function in both neurons and microglial cells (189). Chang et al. present evidence of an association between corticotrophin releasing hormone (CRH) polymorphisms and major depressive disorder (MDD) (190). Kelly and O’Neil have described the influence of glycolysis and tricarboxylic acid cycle function in influencing macrophage polarization to M1 and M2 morphologies, respectively (191). Corcoran and O’Neil have shown that HIF1α, induced by hypoxia, is a primary driver of microglial activation and also of Th17 cell activation, a primary mechanism by which tissue destruction is upregulated in autoimmunity (192).

These examples suggest that, even if changes in neuron-microglial signaling are a neuroimmunological bottleneck, larger therapeutic impacts on the clinical picture in a given patient might be accomplished by addressing other upstream issues, such as those described above that could be immunometabolic, neuroendocrine, or related to hypoxia/oxygenation *via* problems that are circulatory, respiratory, or hematologic. Addressing clinical factors mentioned previously, such as infection, stress, depression, GI dysfunction, poor diet, lack of exercise, and autoimmunity, to the extent present in a given case, may also yield greater impact on the clinical picture. The presence of each of these factors, and their relevance in each case, is a matter of individual clinical discernment. The work of Bredesen suggests that such clinical discernment can be embedded into a structured process that provides for reliably useful clinical outcomes (147).

With regard to the question of whether neuron-microglial dysfunction is a universally applicable feature of cases with CNS involvement, focusing on depression as a well-defined example of a neuroimmunological process involving neuron-microglial interactions, the following papers are useful to consider. Raison and Miller review evidence for two competing views. In one view, inflammation only contributes to depression in a subset of patients. In the other, the inducing influence of inflammation on depression is more widespread, but with vulnerability varying in accordance with the status of a pattern of physiological variables unique to each patient, with each of the variables known to be involved in the etiology of MDD (193). Dooley et al. have described a greater role for inflammation, a key influence on neuron-microglial signaling, in some core features of depression, including exaggerated reactivity to negative information, altered reward reactivity, and somatic symptoms, but has less clear evidence for an involvement in cognitive control (194).

These papers suggest that there is variability from one patient to another in the extent to which neuroimmunological factors influence depression and that there may be variability in the extent to which some features of depression are affected in a given patient. This suggests that the focus on neuron-microglial interaction described in this paper may not apply with equal utility in all cases, or to all features of a given case. Consistent with this view, Kopschina Feltes et al. have suggested that a clinical approach that addresses neuroimmunological factors might have greatest utility in MDD patients with elevated inflammatory cytokines, particularly IL-6, TNF-α and IL-1β, who have been resistant to treatment aimed at monoamine repletion (195, 196).

More research is needed to clarify the above questions and others, with regard to depression, TBI, neurodegenerative conditions, and neuro-autoimmunity, to ensure that in both research and clinical settings, sufficient attention and efforts are focused on areas that could provide significant returns.

## Conclusion

### Neuropsychiatric Disorders Involve Neuroinflammatory and Related Comorbidities

The presence of depression and related neuropsychiatric phenomena in a patient may reflect the presence of an underlying neuroinflammatory state and/or other comorbidities that can affect neuron - microglial interactions, with consequences for neuronal loss and neuro-autoimmunity. The clinician faced with the neuropsychiatric patient may be well served to assess the extent of systemic and neuroinflammation and the other comorbidities discussed and look for potential biological causes that could be instigating or worsening the patient’s neuropsychiatric disorder. It is especially important when a patient presents with neuropsychiatric disorders that the neuropsychiatric nature of the disorder not decrease the clinicians’ inclination to focus on underlying biology but rather increase the clinician’s interest in identifying biological causes of neuroinflammation and related biological factors discussed here.

### Time-Course Issues Matter for Patients With Brain Insults

Once a brain insult develops, the key question is whether it will resolve quickly or progress to a brain in a chronically inflamed state, resulting in greater neuronal loss, greater risk of developing autoimmune responses against brain tissue targets, and expanded loss of function for the patient. The time sequence can yield a cascade of further disease elaboration, including ongoing neuronal loss, epitope spreading, and increasing vulnerability of affected neuronal pools, all yielding further functional loss downstream.

Ongoing case management may need to include a focus on reducing comorbidities that are known to diminish neuronal metabolic integrity, promote neuroinflammation, trigger or perpetuate autoimmune process, increase oxidative burden, or drive other related processes. When expected levels of clinical improvement do not occur, it may be necessary to review the case in light of the known mechanisms by which neuron-microglial interaction can become dysregulated.

## Author Contributions

Conception and authorship of all sections, tables and figures, references, and final edited versioning and approval: SY.

## Conflict of Interest

The author declares that the research was conducted in the absence of any commercial or financial relationships that could be construed as a potential conflict of interest.
